# Extra Virgin Olive Oil from Stoned Olives with Oxygen Supply during Processing: Impact on Volatile and Phenolic Fraction and Sensory Characteristics

**DOI:** 10.3390/foods13193073

**Published:** 2024-09-26

**Authors:** Davide Nucciarelli, Diego L. García-González, Gianluca Veneziani, Stefania Urbani, Luigi Daidone, Sonia Esposto, Agnese Taticchi, Roberto Selvaggini, Maurizio Servili

**Affiliations:** 1 Department of Agricultural, Food and Environmental Sciences, University of Perugia, Via S. Costanzo, 06126 Perugia, Italy; davide.nucciarelli@gmail.com (D.N.); stefania.urbani99@gmail.com (S.U.); luigi.daidone@unipg.it (L.D.); sonia.esposto@unipg.it (S.E.); agnese.taticchi@unipg.it (A.T.); roberto.selvaggini@unipg.it (R.S.); maurizio.servili@unipg.it (M.S.); 2 Instituto de la Grasa (CSIC), Ctra. Utrera, km 1, Edif. 46, 41013 Sevilla, Spain; dlgarcia@ig.csic.es

**Keywords:** virgin olive oil, volatile compounds, phenols, oxygen influence, sensory quality

## Abstract

The improvement of the extra virgin olive oil (EVOO) extraction process involves the proper management of endogenous enzymes of the olive fruit and all the technological conditions that can affect their activities. Coratina and Peranzana cultivars were processed to assess the influence of different technologies for fruit breaking (crushing and stoning) with and without controlled oxygen addition during this critical phase. The study of volatile compounds revealed that the enzymes that are responsible for their genesis during the technological process were significantly affected by oxygen addition in both the systems of fruit crushing. The results from the stoning technology proved that the quality improvement was a consequence of the prevention of the seed breaking and the oxidation catalyzed by the olive stone enzymes. In Peranzana EVOOs, it was possible to increase the aldehyde concentration up to 97% using stoning technology with a 0.2 L/min oxygen addition compared with traditional crushing. At the same time, non-significant reductions in phenolic compounds were detected when comparing crushing and stoning with and without the addition of oxygen, and similar trends were observed for the two studied cultivars. The sensory analyses confirmed the differences in phenolic and volatile composition detected in the EVOO samples.

## 1. Introduction

In recent years, many studies have been conducted to improve the quality of EVOO. Some technologies that come from other food production sectors or different industries are now being tested in the virgin olive oil (VOO) extraction sector with the aim of improving extraction process technology. These innovations enhance the quality of oils and improve the efficiency of the process. On the other hand, knowledge of the raw material and all the variables that affect it during processing are critical for an optimized extraction process. Thus, better control of the extraction parameters is currently an indispensable strategy that should be implemented in all olive oil production industries. In particular, new technological innovations are trying to modify the entire olive oil sector in the direction of high-quality production by improving its shelf life, nutraceutical value, and sensory profile [1,2,3,4,5,6,7]. The aim of these innovations especially focuses on managing enzyme activity to increase the production of volatile compounds and avoid the oxidation of phenols or increase their concentration in VOOs [8,9,10,11]. Another aim of these innovations is to increase extraction yields and improve the general efficiency of extraction plants [12,13,14,15]. An important factor that affects process innovations is that VOO production must use physical or mechanical methods only according to EU regulations [16]. Previous studies have focused on the influence of oxygen during the crushing phase [17,18,19,20], highlighting the important role that oxygen plays in the lipoxygenase (LOX) pathway responsible for volatile compound production. Currently, it is well known that oxygen is a limiting factor for the activity of the LOX enzyme [20,21,22]. On the other hand, adding oxygen to the VOO extraction process could be a problem due to the oxidation of phenols [12,23,24]. As we know from previous research, the phenols in the oil are oxidized by enzymes and oxygen [17,24]. Therefore, prevention of this oxidation can be managed by fine optimization of the presence of oxygen and major control of the presence and/or activity of the enzymes [17,22,25]. Moreover, some studies have focused on the different modalities of fruit breaking, such as traditional crushing, differentiated crushing, and stoning [2,8,26,27,28]. Differentiated crushing coupled with thermal conditioning can produce very high-quality olive oil [29]. In fact, this emerging technology could lead to a new extraction system that implements stoning at the same time to maintain good extraction yields. This system could provide advantages from the point of view of not only quality but also of low consumption of machine parts that normally work in contact with the stone fragments. These components, such as progressive cavity pumps and decanter screws, cause considerable wear and tear due to the presence of stone fragments [30,31]. Thus, it is also possible to use other volumetric pumps that are easier to clean and/or have longer usage due to the use of stainless steel components. The use of stoning, which can involve stones of a considerable volume, allows improving the capacity of the plant. At the same time, using stoning in production also permits the production of natural heating fuel from a renewable source [32,33,34,35], since unlike normal stone, this material with seeds also contains oil [36]. Furthermore, for stoning technology, another important advantage is that the final subproduct of the process is an easily usable feed for animals that contains many antioxidant compounds and a low content of lignin for better digestibility [37,38]. Several studies have demonstrated that stoning technology results in better sensory quality than crushing [26,27,28]. This fact depends on the absence of crushing or cutting of seeds that contain many enzymes that affect the final quality of VOO [2,3,4,5,6,7,8,9,10,11,12,13,14,15,16,17,18,19,20,21,22,23,24,29]. In particular, the seeds contain polyphenoloxidase (PPO) and peroxidase (POD), which are responsible for phenol oxidation [25,39]. In this context and considering the results of a previous work [22], it was important to investigate whether the effect of oxygen addition also depends on the system of fruit breaking. Stoning was abandoned due to problems related to yield extraction. In fact, traditional crushing leads to different fragments of stone with a homogeneous diameter distribution, which improves the breakdown of pulp olive cells releasing a higher amount of oil droplets and also improves oil drainage in decanter centrifuges, increasing the oil extractability [26,27,40]. Even if the crushing systems underwent significant innovations concerning the biological characteristics of the fruit, especially with a reduction of the breaking impact on olive seed minimizing the release of oxidative enzymes in the olive paste [41], stoning was a better method for fruit breaking [27,28]. In addition to oxidation and the concentration of volatile compounds, all oils produced using stoning technology have a relatively high concentration of volatile compounds that are responsible for the green fruity aroma [29,42]. In this case, flash thermal conditioning also improves the activity of the enzymes [11]. In fact, it is important to note that a remarkable amount of PPO is still present in the olive paste, and its presence is inherent to the extraction process [25,43]. This means that its activity must be controlled during the process. Thus, even if the quality improvement resulting from stoning is relevant, it is better to consider the possible negative effects associated with changes in enzyme activity simultaneously. Tubular heat exchangers have also been tested for their ability to modulate enzyme activities in pitted olive paste [29]. A similar application of heat transfer coupled with stoning was also described by Amirante et al. [44], who reported improvements in oil quality. Other technologies, such as ultrasound, high vacuum malaxation, and pulsed electric field, can help stoning due to their ability to increase oil extractability in the extraction phase using decanters [25,26,27,28,39,40,41,42,43,44,45]. Considering that all these technologies improve their efficiency according to the moisture of the paste, the stoning operation allows for an increase in the paste moisture and a decrease in the texture hardness due to the elimination of the woody fraction and the nut kernel. This combination of technology could permit good yield extraction in addition to a very high-quality product.

Considering the necessity of improving the sensory and nutraceutical characteristics of VOOs through the use of current innovation advances, this work aimed to test the effect of oxygen combined with different kinds of olive breaking. Even if it is already known that stoning increases the intensity of fruity attributes [26,27,28], this work aimed to prove that oxygen in the stoning phase can increase the activity of LOX enzymes, and this activity can be reflected in an increase in the concentration of volatile compounds that are responsible for fruity attributes, as normally occurs in the crushing phase [19,20,24]. Considering that the machine is completely different from a crusher and the chamber in which fruit crushing occurs (closer to 0.1 m^3^ instead of 0.01 m^3^), oxygen under these conditions may have an impact on the activity of the different enzymes that are included in the pool of the LOX pathway. This work also aimed to investigate the effect of oxygen addition during this phase and how this possible improvement of the machines could be adapted to different cultivars due to their biological behavior, which is influenced by genetic characteristics. This study is a continuation of one that was conducted by modeling the flow of oxygen in addition to the crushing phase [22]. Through the use of the same extraction pilot plant, the best of four oxygen concentrations was identified, after which different systems of fruit breaking were investigated to determine the combined effect of these systems with and without oxygen addition.

## 2. Materials and Methods

### 2.1. Materials

Phenyl alcohols (3,4-DHPEA and *p*-HPEA) were acquired from Fluka (Milan, Italy) and Cabru s.a.s. (Arcore, Milan, Italy), respectively. Hydrophilic phenols (secoiridoids derivatives and lignans) were obtained as described by Selvaggini et al. [46]. All the other chemical compounds and solvents were supplied by Merck (Merck KGaA, Darmstadt, Germany).

### 2.2. Extraction Plant Equipped for Oxygen Addition during Crushing or Stoning

EVOO samples were produced by using an industrial pilot plant scale with a working capacity of 300 kg/h in the Research Unit of Food Technologies in the Department of Agricultural, Food and Environmental Science at the University of Perugia.

Before the olives were crushed or stoned, they were cleaned of leaves, wood, rocks, soil, and all the foreign matter. The olives were then added to the washing section. After the first part of cleaning and washing, the olives were ready to be processed starting with a crusher or a pitter. The plant was equipped with a system for supplying oxygen during the first phase of fruit breaking (crushing or stoning). The concentration of oxygen must be related to the extraction plant capacity and, in particular, to the crushing flow rate. In all the tests, the flow of the olives at the inlet of the crusher was nearly 640 kg/h. Thus, the quantity of oxygen was 0.019 L for each kg of olives at a flow rate of 0.2 L/min. The oxygen was put inside the crusher chambers using a 1/4″ ball valve 170 mm from the axle of the crusher inside the zone of the sieve. For the pitter, the oxygen inlet was placed close to the olive inlet in the stoning chamber. The suction effect of the pitter due to the rotation of the rotor directly injected the flow of oxygen inside the pitter’s chamber. The oxygen was available at the moment when the fruit was breaking, and the flow started when the olives entered the stoning chamber. The same quantity of oxygen supplied to the crusher was added to the pitter at the same working capacity as the olives because the flow of the olives was fixed by the speed of the elevator feed screw. The pitter used was a 700 kg/h capacity (Alfa Laval, Tavarnelle Val di Pesa, Italy). The crusher used was an FR 350 hammer crusher (TEM, Tavarnelle Val di Pesa, Italy).

The flow was measured by using a flow meter with a sensitivity of ±0.05 L/min, which is usable only for oxygen gas (TEKNOM a/m 145, Figline e Incisa Valdarno, Italy).

After crushing or stoning, the olive paste was pumped into a Visco Line Heat Exchanger (Alfa Laval Corporate AB, Monza, Italy) set at 18 °C. Then, the olive paste was transferred into a 200 kg capacity gas insulated malaxer (TEM, Tavarnelle Val di Pesa, Italy) set at 25 °C for 30 min of malaxation.

After malaxation, the oil was separated from the paste using a 300 kg/h capacity decanter centrifuge (TEM 200 system, Tavarnelle Val di Pesa, Italy) at a flow rate of 200 kg/h of olive paste and was finally purified from a large portion of the water, solids, and colloids by a centrifuge vertical separator (Alfa Laval UVPX 305 AGT 14; Alfa Laval, Tavarnelle Val di Pesa, Italy). The withdrawal of the sample was made at the half time of the malaxer discharge to avoid contamination with the previous EVOOs for each trial. For each trial the tests were replicated two times. The oil samples were filled into half-liter glass bottles, sealed, and stored under darkness condition at 13 °C until analyses.

The maturity indices of the olives were 1.08 for Peranzana and 0.87 for Coratina, as measured according to the methods of Beltrán et al. [47]. The samples were coded according to cultivar (Peranzana, PE; Coratina, COR), processing (crushing, CR; stoning, ST), and oxygen supply (O_2_).

### 2.3. EVOO Chemical Analysis

#### Legal Quality Parameters

The analyses relating to acidity, peroxide value, and spectrophotometric constants (absorbance in the ultraviolet range, K232, K270, and ΔK) were carried out according to current EU regulations [16].

### 2.4. Phenolic Compounds

The extraction of VOO phenolic compounds was performed in accordance with Taticchi et al. [48]. The HPLC analyses of the phenolic extracts were conducted according to Selvaggini et al. [49] with a reversed-phase column using an Agilent Technologies system Model 1100 (Agilent Technologies, Santa Clara, CA, USA), which was composed of a vacuum degasser, a quaternary pump, an autosampler, a thermostated column compartment, a DAD, and a fluorescence detector (FLD). The C18 column used in this study was a Spherisorb ODS-1 250 mm × 4.6 mm with a particle size of 5 μm (Waters, Milford, MA, USA); the injected sample volume was 20 μL. The mobile phase was composed of 0.2% acetic acid (pH 3.1) in water (solvent A)/methanol (solvent B) at a flow rate of 1 mL/min, and the gradient changed as follows: 95% A/5% B for 2 min, 75% A/25% B in 8 min, 60% A/40% B in 10 min, 50% A/50% B in 16 min, and 0% A/100% B in 14 min; this composition was maintained for 10 min and then returned to the initial conditions and equilibration in 13 min; the total running time was 73 min. The quantitative evaluation of phenols was carried out by means of single calibration curves for each compound [the dialdehydic forms of decarboxymethyl elenolic acid linked to hydroxytyrosol (3,4-DHPEAEDA or oleacein) and to tyrosol (p-HPEA-EDA or oleocanthal), 3,4-(dihydroxyphenyl)ethanol elenolic acid (3,4-DHPEA-EA or an isomer of the oleuropein aglycon), and p-(hydroxyphenyl) ethanol elenolic acid (p-HPEA-EA or ligstroside aglycon) with a range from 5 to 800 mg, tyrosol (p-HPEA), hydroxytyrosol (3,4-DHPE), (+)-1-acetoxypinoresinol, (+)-pinoresinol and vanillic acid with a range from 0.1 to 50 mg)], and the results are expressed as mg/kg of oil. Data were expressed as mg of phenolic compounds per kg of EVOO.

### 2.5. Volatile Compounds

Evaluation of volatile compounds in VOOs was performed by headspace solid-phase microextraction, followed by gas chromatography−mass spectrometry (HS-SPME/GC-MS, Agilent Technologies, Santa Clara, CA, USA), according to the method of Taticchi et al. [48]. Three grams of oil was mixed with 2-methylpropyl acetate as internal standard at the concentration of 9.8 mg/kg. To sample the headspace volatile compounds, solid-phase microextraction (SPME) was applied as follows: all of the vials were held at 35 °C for 10 min, and then the SPME fiber (a 50/30 μm 2 cm long DVB/Carboxen/PDMS, Stableflex; Supelco, Inc., Bellefonte, PA, USA) was exposed to the vapor phase for 30 min to sample the volatile compounds. The gas chromatography-mass spectrometry analyses (GC-MS) was performed using an Agilent Technologies GC 7890B with “Multimode Injector” (MMI) 7693A coupled to a single quadrupole MSD mod. 5977B using an EI Extractor (XTR) source (Agilent Technologies, Santa Clara, CA, USA); a thermostated PAL3 RSI 120 autosampler equipped with a fiber conditioning module and an agitator (CTC Analytics AG, Zwingen, Switzerland) was also employed. For the chromatographic conditions used for the analysis of volatile compounds, see Taticchi et al. [48] Volatile compounds were identified by comparing their mass spectra and retention times with those of authentic reference compounds and with spectra in the NIST 2014 mass spectra library. The quantitation of the volatile compounds was performed using the calibration curves (internal standard method) for each compound, and the results are expressed as µg/kg of oil.

### 2.6. Sensory Analysis

All the olive oil samples were submitted to sensory analysis following the instructions described by Venezaini et al. [22] based on Commission Delegated Regulation (EU) 2022/2104 [16] and the International Olive Council (COI). Twenty-one attributes (“deep green”, “moss green”, “yellow–green”, “yellow”, “fatty”, “sweet”, “bitter”, “pungent”, “fruity”, “herbaceous”, “artichoke”, “hay”, “green apple”, “floral”, “tomato”, and “almond”), for the visible assessment, olfactory, and gustative sensory notes, were used to perform the sensory analyses of EVOO flavour with the addition of some attributes to individuate the off-flavors characteristics (“earthy”, “winey-vinegary”, “rancid”, and “fusty”). A spider graph was used to show the sensory profile of the different olive oils, elaborating the data with Principal Component Analysis (PCA).

### 2.7. Statistical Analysis

The analysis of variance (ANOVA) was carried out by using SigmaPlot V.12.3 (Systat Software Inc., San Jose, CA, USA) to evaluate the statistical differences among the data obtained by control and the different experimental tests. Tukey’s test (*p* < 0.05). was used to process the data.

The multivariate analyses conducted with the PCA technique were performed with Panel Check version 1.4.2 software (Nofima Mat, Tromsø, Norway).

## 3. Results and Discussion

The oil yield was influenced by the stoning process, as reported by different authors [26,27,40], with a reduction of 1.2% and 2.2% for Coratina and Peranzana cultivar, respectively. On the contrary, the oxygen supply during the crushing phase did not obviously show any impact on oil extractability.

### 3.1. Legal Quality Parameters

The extracted EVOOs were analyzed based on the legal quality parameters to obtain basic information on the quality of the oils. The objective was to determine whether the samples produced by the addition of oxygen during the crushing or stoning phases could be classified as the EVOO category according to the EU regulations [16]. Table 1 shows the values for free acidity, peroxide value, K232, K270, and ΔK for the Peranzana and Coratina EVOOs. The results showed that the different technologies used for fruit breaking, combined with an oxygen supply, allowed the production of virgin olive oil within the extra virgin category. In fact, when comparing the values of the EVOOs produced by different systems of fruit breaking and oxygen addition, no significant differences were observed for all the legal quality parameters of the different samples (Table 1) that belong to the two different cultivars analyzed.

### 3.2. Phenolic Compounds

Table 2 shows the phenolic composition of the oils produced by using different fruit-breaking modalities. The addition of oxygen at 0.2 L/min did not significantly affect the total phenol concentration. This occurred for both cultivars and was independent of the fruit-breaking system used. This result confirmed once again that it is possible to increase the concentration of oxygen during fruit breaking without significantly decreasing the total concentration of phenol compounds. For all the samples produced using stoning technology (samples coded as ST in the table), the total phenol concentration increased significantly in comparison with that of the samples produced using crushing technology, which agrees with results from previous works [42,50,51,52]. For the cultivar Peranzana, no significant differences were detected in the concentrations of total phenols, oleuropein derivatives, ligstroside derivatives, or lignans. In fact, no significant differences were found when oxygen was added for the different fruit-breaking modalities tested. These results were in accordance with a previous study on the oxygen concentration supplied during the crushing phase [22]. Regarding the individual compounds, a slight influence likely due to oxygen addition was detected only in the 3,4-DHPEA-EA concentration, which showed a significant decrease of approximately 25% between the control samples (PE CR, traditional crushing) and the samples produced by crushing with an oxygen addition of 0.2 L/min (PE CR-O_2_). For the same compound, no significant differences were detected between the oils produced by stoning (PE ST) and those produced by stoning with oxygen addition (PE ST-O_2_). This difference between the PE CR and the PE CR-O_2_ groups could be related to the greater availability of oxygen and the greater capacity for oxidation of endogenous enzymes in the olive seeds, as reported in previous works [43,53,54,55]. In this cultivar, the concentrations of the remaining phenolic compounds were similar or lower, although these differences were less relevant than those of 3,4-DHPEA-EA.

For the Coratina cultivar, the same trend was observed for the Peranzana cultivar in terms of total phenol concentration, oleuropein derivatives, ligustroside derivatives, and lignans. In contrast to the Peranzana cultivar, there was no difference in 3,4-DHPEA-EA, which showed no significant differences among the samples produced by all the different modalities of fruit breaking. These results once again confirmed that the cultivar dependence in this case was slight. Instead, a common behavior was observed even if the cultivars processed were strongly different in terms of phenol concentration. Further investigations should be performed in the future to better understand cultivar influence in relation to the technology type used for fruit breaking and in association with other important factors. Thus, the influence of the maturity index should be evaluated in terms of its effect on different technologies used for fruit breaking in combination with oxygen addition. This knowledge could be important for better optimization of oxygen addition with a comprehensive approach that considers the effect of oxygen addition for specific cases of crushing or stoning phases, cultivars, and maturity indices for minimizing the oxidation of phenols and fatty acids. In addition, considering the previous knowledge, it could also be important to identify the right temperature of malaxation for each combination of cultivar and maturity index. The concentration of phenols also depends on the temperature of malaxation [46]. The management of all the factors that can affect the concentration of phenols could aid in determining the parameters of the production process.

### 3.3. Volatile Compounds

Table 3 shows the volatile compound data obtained for the EVOOs produced in this study. The results proved that the stoning technology modified the volatile concentration of the oils of both cultivars. Thus, the addition of oxygen at 0.2 L/min modified the volatile composition of the oils produced from Coratina and Peranzana olives, as investigated in previous work with different cultivars [22]. Independent of the use of oxygen, for the cultivar Peranzana, the oil produced using stoning technology had a higher concentration of volatile compounds, namely, C_5_ and C_6_ aldehydes and esters, and at the same time had a lower concentration of C_5_ and C_6_ alcohols. These results confirmed the improvements in the oil sensory profile associated with stoning technology, as reported in several previous studies [26,27,28]. In this study, the effect of oxygen addition in the stoning phase was investigated to better understand the behavior of the enzymes responsible for volatile production during the extraction of oil from olives. In particular, LOX pool enzymes are responsible for the production of volatile compounds that determine the aroma profile of VOO [4,56,57,58,59]. According to the results described above, the presence of oxygen and the temperature of thermal conditioning immediately after the stoning phase were likely more favorable conditions for the activities of the LOX pool enzymes [56,60,61]. One of the most evident results was the increase in the concentration of the sum of the C_5_ and C_6_ aldehydes in the Peranzana samples. There was an increase for every step in terms of improving the breaking modality: a 47% increase in crushing with the addition of oxygen (PE CR-O_2_) compared to traditional crushing (PE CR), a 21% increase in stoning (PE ST) compared to crushing with the addition of oxygen (PE CR-O_2_), and an 11% increase in stoning with oxygen addition (PE ST-O_2_) compared to stoning (PE ST). The difference in concentration between traditional crushing (PE CR) and stoning with oxygen addition (PE ST-O_2_) was 97%. The concentrations of (*E*)-2-hexenal followed the same trend as the total concentrations of the C_5_ and C_6_ aldehydes. In fact, it represented the most abundant compound in these clusters of volatiles. (*E*)-2-hexenal is normally related to the sensory attribute of “herbaceous”, as several previous studies have reported [57].

The results also revealed interesting changes in the concentration of esters when the values were compared among the samples produced by using different modalities of fruit breaking. The trend for esters was similar to that observed for the C_5_ and C_6_ aldehydes, with some minor differences. The concentration of esters increased for every step of changing the fruit-breaking modality except for the last one. In fact, the concentration of esters increased in the PE CR-O_2_ compared with the PE CR and in the PE ST compared with the PE CR-O_2_. In particular, there was an increase in the ester concentration in the PE CR-O_2_ sample in comparison with the oil produced with traditional crushing. The concentration of these compounds in the oil contributes to floral and sweet fruit olfactory sensations in the sensory profile in accordance with other factors [57,62,63]. Considering the industrial pilot plant scale, these changes in EVOO production may lead to satisfactory results in terms of the biochemical processes that contribute to the sensory quality of the oil.

For the Coratina cultivar, for the sum of the C_5_ and C_6_ aldehydes, the samples produced by adding 0.2 L/min of oxygen during crushing did not show a significant difference compared with the samples produced by stoning with oxygen addition. However, there is a significant difference among the samples produced by traditional crushing and those produced by stoning with no added oxygen. No difference was detected for the concentrations of esters. These compounds normally have lower concentrations in Coratina oils than in Peranzana oils [29,60]. Thus, this difference could depend on the genetic characteristics of the olives [60]. A previous study on other cultivars showed an increase in the volatile concentration when crushing was immediately followed by thermal conditioning at 18 °C [11]. In the present study, thermal conditioning was also coupled with stoning (PE ST, COR ST) and stoning with oxygen addition (PE ST-O2, COR ST-O2). For each of these improvements, the higher concentrations of C5 and C6 aldehydes in the Peranzana cultivar than in the Coratina cultivar, as shown above, could be explained by the favorable conditions for the activity of the LOX pool enzymes due to the presence of oxygen [64,65,66] and the temperature of thermal conditioning immediately after stoning [11,44].

### 3.4. Sensory Analysis

The impact of two typologies of olive breaking and oxygen supply during processing was also evaluated in the sensory profile of EVOO. The sensory analysis was performed using a quantitative descriptive analysis form in which the trained tasters reported the intensity of various descriptors on unstructured scales.

**Figure 1:**
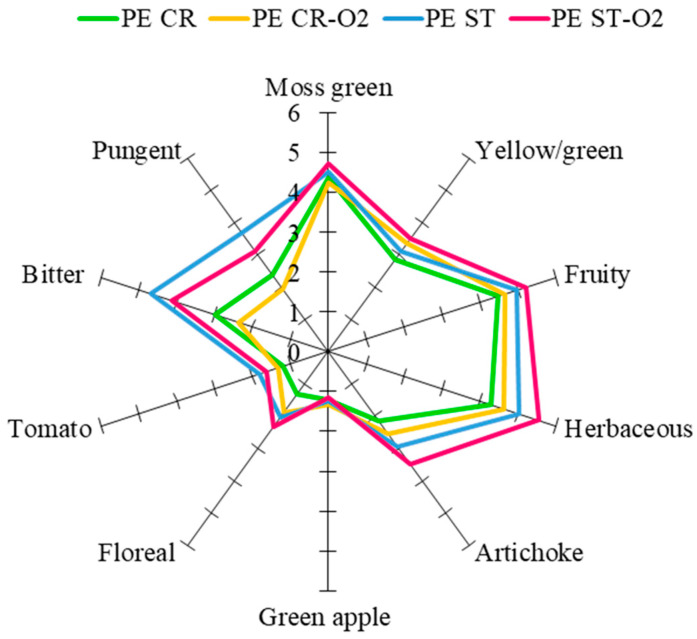
Sensory profiles of Peranzana virgin olive oils obtained with four different modalities of fruit breaking: crushing (PE CR), crushing including 0.2 L/min of O_2_ addition (PE CR-O_2_), stoning (PE ST), and stoning including 0.2 L/min of O_2_ addition (PE ST-O_2_). Results show significant differences (*p* < 0.05) among processes.

The sensory analyses carried out on the EVOOs partially confirmed the results obtained by instrumental analysis in volatile and phenolic composition. The olive breaking by traditional hammer crusher (CR) and stoning process (ST) with and without oxygen addition (0.2 L/min) had an impact on the sensory descriptors of all the EVOOs tested. There were differences between the EVOOs obtained by hammer crusher (CR) and the EVOOs produced by the stoned process (ST) with oxygen addition. In both cultivars, the main differences detected were in the intensity of the fruity sensory note, related to the activity of the LOX pathway, that produces C_5_ and C_6_ aldehydes during processing. Table 3 shows an increase of the sums of the C_5_ and C_6_ aldehydes that was approximately 42% in the EVOOs produced by stoning and oxygen supply in comparison with the control. The C_5_ and C_6_ aldehydes are associated with green grass sensory notes, such as “herbaceous” [4,66,67]. Figure 1 shows the results of the sensory analyses of EVOOs from Peranzana cv and shows significant differences (*p* < 0.05) for the “pungent”, “bitter”, and “herbaceous” attributes. For the olfactory sensory notes, an increase in the “herbaceous” attribute was observed in relation to the oxygen addition in both types of olive breaking applied. It should be noted, however, that the intensity of the “herbaceous” note was greater for all the EVOOs obtained from stoned olive pastes. The results are in agreement with the concentration of the C_5_ and C_6_ aldehydes, (*E*)-2-hexenal in particular, reported in Table 3. As reported in previous papers [4,66,68], the (*E*)-2-hexenal can be considered an “impact compound” for the “herbaceous” grass sensory note in EVOO. Considering the “artichoke” and “fruity” attribute, the same trend as the “herbaceous” attribute was detected. As reported above, the cluster of attributes “green grass”, “fruity”, “almond”, and “artichoke” was related to the generation of the C_5_ and C_6_ aldehydes in EVOO during processing [69].

The “floral” sensory note demonstrates the same trend of “herbaceous” and “fruity” attributes and was close related to the esters, produced by the alcohol-acetyl-transferase activity [66,68,70] that, as reported in Table 3, show the same tendency. The esters increase was affected by the oxygen addition, which is a co-factor of the initial oxidation of linolenic and linoleic acids [68,71].

The gustative attributes, such as “pungent” and “bitter”, were positively affected by the stoning process while showing a negative relationship with the oxygen addition during processing. As extensively reported in previous papers, these two attributes are correlated with the concentration of secoiridoids aglycon in EVOO [41,62,72]. In particular, the bitterness is mainly related to the presence of oleacein (3,4-DHPEA-EDA), and the pungency sensory note is mainly associated to the presence of olechantal (p-HPEA-EDA) [41,72,73,74,75]. As reported in Table 2, the phenolic compounds show a significantly greater concentration in EVOOs produced by stoning than in the EVOOs obtained by hammer crusher. This difference was also observed in the sensory profiling reported as a spider graph in Figure 1 and was associated to the distance between the values of the “bitter” and “pungent” attributes.

**Figure 2:**
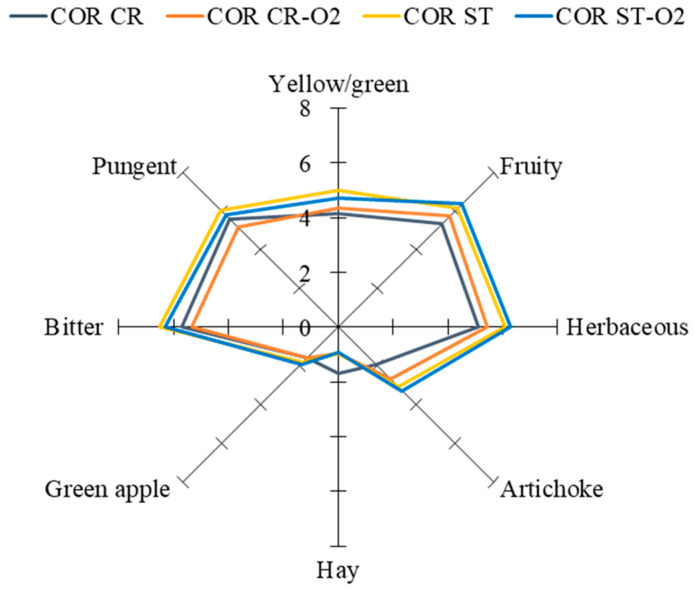
Sensory profiles of Coratina virgin olive oils obtained with four different modalities of fruit breaking: crushing (COR CR), crushing including 0.2 L/min of O_2_ addition (COR CR-O_2_), stoning (COR ST), and stoning including 0.2 L/min of O_2_ addition (COR ST-O_2_). Results show significant differences (*p* < 0.05) among processes.

The spider graph of the sensory analyses of the Coratina EVOOs, reported in Figure 2, show significant differences (*p* < 0.05) for the “fruity”, “bitter”, and “herbaceous” sensory notes. As already observed for the sensory profiles of Peranzana EVOOs, the highest values of “fruity”, “herbaceous”, and “artichoke” were detected in the oils produced by stoning in combination with oxygen supply and can be associated with the concentrations of the C_5_ and C_6_ aldehydes shown in Table 3. In particular, (*E*)-2-hexenal was responsible for green grass as explained above. Unlike the Peranzana EVOOs, the values of the “floral”, “tomato”, and “green apple” sensory attributes were not associated with the Coratina EVOOs. The “bitter” and “pungent” attributes showed higher values in comparison with the Peranzana EVOOs due to the difference in the concentration of phenols, associated with the cultivar effect, that are responsible for those gustative sensory perceptions.

**Figure 3:**
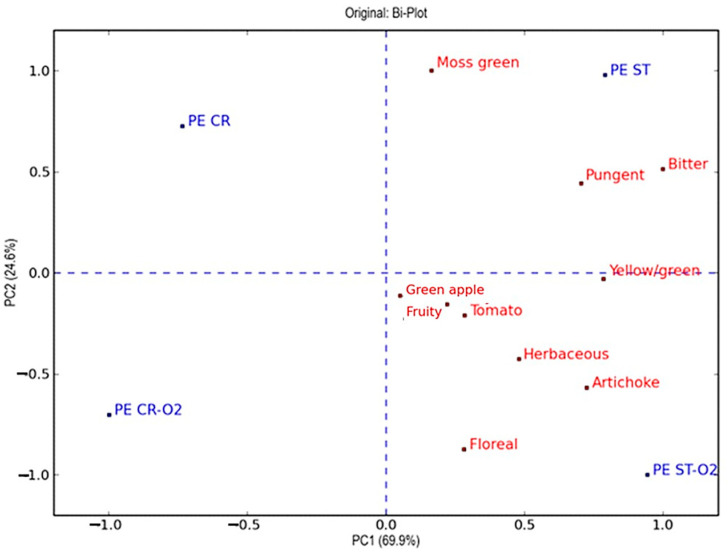
Representation of objects and variables in the Bi-Plot that resulted from the principal component analysis (PCA) on the plane of the two principal components (PC1 and PC2) relating to the results obtained through sensory evaluations of all virgin olive oils of cv. Peranzana (objects) and attributes (variables) evaluated by the panel test. The variables and objects are marked in red and blue, respectively.

**Figure 4:**
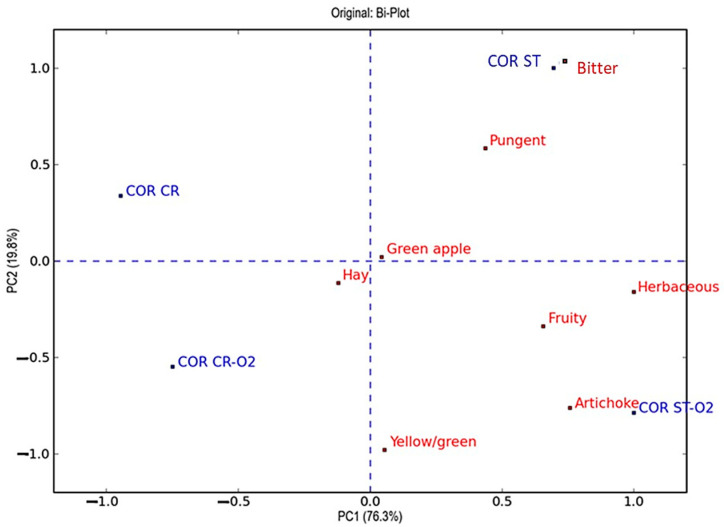
Representation of objects and variables in the Bi-Plot that resulted from the principal component analysis (PCA) on the plane of the two principal components (PC1 and PC2) relating to the results obtained through sensory evaluations of all virgin olive oils of cv. Coratina (objects) and attributes (variables) evaluated by the panel test. The variables and objects are marked in red and blue, respectively.

To better explain the differences in the sensory profile, the PCA was carried out with the scores obtained by the panellists. The PCA plots, reported in Figure 3 and Figure 4, confirmed that the EVOOs obtained with hammer crusher and stoning crushing show different intensities of the “fruity” attribute. Therefore, the lowest values correspond to the EVOOs produced using the traditional hammer crusher, followed by the oils obtained with the oxygen addition with the same crusher, followed by the EVOOs produced by destoning, and finally by those obtained by combining destoning and oxygen addition.

The score plot of the Peranzana cultivar, shown in Figure 3, explained 94.5% of the variance with two components. The first principal component explained 69.9% of the total variance and differentiated the samples according to the olive-breaking process, traditional hammer crusher, and stoning. For the first component, the olive-breaking procedure applied clearly affected the sensory profiles of the EVOOs: on one side were the EVOOs produced by the traditional hammer crusher, and on the other side were those obtained by stoning. The sensory descriptors that discriminated the samples among the two components, in order of relevance, were “pungent”, “bitter”, “yellow/green”, “artichoke”, and “herbaceous”. The differences between the EVOOs obtained by traditional crushing and those produced by stoning were identified mainly for these attributes that, as previously pointed out, are related to the concentration of phenols and volatile compounds such as the C_5_ and C_6_ aldehydes. The second component, which explained 24.6% of the total variance, shows a clear discrimination between the EVOOS produced using traditional crushing or stoning with and without the oxygen addition. In fact, the addition of oxygen affected the separation of the samples along the second component.

The PCA score plot related to the EVOOs produced from the cultivar Coratina, reported in Figure 4, explained 96.1% of the total variance with two principal components. The first component, which explained 76.3% of the total variance, discriminates the samples in two clusters according to the olive-breaking process, traditional hammer crusher and stoning, respectively. For the olfactory sensory notes, the discrimination along the first component was explained by the intensity of “herbaceous”, “artichoke”, and “fruity” sensory attributes that were higher for the EVOOs produced by stoning. As already pointed out for the oils obtained from the Peranzana cultivar, the intensity of these sensory notes is associated with the concentrations of the C_5_ and C_6_ aldehydes [20], which, as reported in Table 3, were higher for the EVOOs produced by stoning. For the gustative sensory attributes, the discrimination of EVOOs was associated with the intensity of “bitter” and “pungent” sensory attributes, which were higher for the EVOOs produced by the stoning process, in agreement with the differences in the phenolic concentration reported in Table 2. Relative with the second principal component, which explained 19.8% of the total variance as observed for the EVOOs produced by Peranzana cultivar, the samples were discriminated according to the oxygen addition in the pastes during processing. The samples discrimination was explained according to the intensity gustative attribute, such as “bitter” and “pungent”, and by the “artichoke” attribute for the olfactive sensory notes.

## 4. Conclusions

This study confirmed that the use of stoning technology in the extraction process of EVOO can improve quality in terms of the concentrations of volatiles and phenols and the legal quality parameters. This result also confirmed that in the seed, there are several biochemical activities, mainly represented by large amount of peroxidase, that can decrease the final quality of the oil produced. The results showed that the oxygen activity depends on the cultivar, but the influence of oxygen is also dependent on the system of fruit breaking. This work showed that it is possible to improve the sensory quality without compromising the antioxidant activity due to the concentration of phenols. From the results, it could be concluded that it is important to provide the enzymes with the best conditions to promote the activity that improves quality, based on the study of the enzymatic biochemical behavior. In previous works, four possible concentrations of oxygen and how their effects differed depending on the cultivar were studied. For further research, it would be difficult to perform a general screening for all the cultivars, as this would involve many industrial plants with a large quantity of olives. The significant effects of oxygen and the olive-breaking type are consistent between the two cultivars used in this study. Considering the relatively low concentration of oxygen added in the crushing or stoning phases, this technological update to the plant equipment could be easily implemented in an existing industrial plant.

In all the systems, the addition of oxygen during fruit crushing showed a significative positive effect by increasing the concentration of volatile compounds, particularly in the Peranzana oils produced by the stoning method, in which the greatest increase in the C_5_ and C_6_ aldehydes, which are responsible for the green fruity aroma, was detected. As previously discussed, even if the stoning method is the best method of fruit breaking, many olive oil mills cannot use this system because the yield extraction is negatively affected. However, the use of stoning is recommended in cases in which an increase in product quality justifies a higher selling price. This better quality and added value must offset or exceed the cost of yield loss, compared to a process with traditional crushing, along with the advantages from the process point of view. Furthermore, process-related advantages such as less wear and tear on pumps, crushers, and decanters, and higher production capacity, must also be considered. All these conclusions must be related to industrial-scale process production, in which the economic sustainability of the process is highly important. If the final consumer understands and appreciates the importance of product quality from a sensory point of view, it could be possible to apply these improvements in milling plants. However, it should be emphasized that a similar high-quality EVOO can be obtained with the correct management of technologies, for example, by using differentiated crushing, which is able to contain the negative oxidation activity of PPO and POD and to reduce the breakdown of olive seed tissues followed by a rapid thermal conditioning of olive paste at 18 °C to improve the LOX activity. The aims of further studies will be to identify the behaviors of the biological variables and the biochemical processes related to oil extraction from olives; then, based on these data, the process can be managed to obtain a product of the desired quality.

## Figures and Tables

**Table 1:** Legal quality parameters determined in the produced virgin olive oils from Peranzana (PE) and Coratina (COR) cultivars, obtained with traditional crushing (CR) and stoning (ST), with (O_2_) and without oxygen supply during extraction.

Parameters	Peranzana				Coratina			
	PE CR	PE CR-O2	PE ST	PE ST-O_2_	COR CR	COR CR-O_2_	COR ST	COR ST-O_2_
Acidity (% oleic acid)	0.28 ± 0.01a	0.28 ± 0.01a	0.28 ± 0.01a	0.27 ± 0.01a	0.23 ± 0.01a	0.23 ± 0.01a	0.25 ± 0.01a	0.24 ± 0.01a
Peroxide value (meq O_2_/kg of oil)	5.93 ± 0.15a	6.2 ± 0.3a	5.93 ± 0.45a	6.07 ± 0.21a	3.27 ± 0.46a	2.7 ± 0.52a	3.2 ± 0.52a	2.73 ± 0.12a
K232	1.74 ± 0.06a	1.74 ± 0.04a	1.71 ± 0.01a	1.73 ± 0.02a	1.64 ± 0.07a	1.58 ± 0.07a	1.54 ± 0.05a	1.54 ± 0.15a
K270	0.18 ± 0.02a	0.18 ± 0.01a	0.18 ± 0.001a	0.19 ± 0.01a	0.16 ± 0.02a	0.17 ± 0.01a	0.18 ± 0.01a	0.18 ± 0.03a

Note: Values are the mean of two determinations in two independent trials ± the standard deviation. Different letter a in the row indicate that results are statistically different among theses for each different cultivar (*p* < 0.05). The ΔK was lower than 0.01 in all cases.

**Table 2:** Composition of phenolic compounds (mg/kg) determined in the virgin olive oil samples from Peranzana (PE) and Coratina (COR) cultivars, obtained with traditional crushing (CR) and stoning (ST), with (O_2_) and without oxygen supply during extraction.

Phenolic Compounds	Peranzana				Coratina			
	PE CR	PE CR-O_2_	PE ST	PE ST-O_2_	COR CR	COR CR-O_2_	COR ST	COR ST-O_2_
Hydroxytyrosol	1.0 ± 0.1a	1.2 ± 0.5a	1.4 ± 0.8a	3.3 ± 1.8a	2.0 ± 0.5ab	1.6 ± 0a	2.1 ± 0.4ab	2.5 ± 0.4b
Tyrosol	1.8 ± 0.3a	1.8 ± 0.1a	1.8 ± 0.2a	2.2 ± 0.3a	2.4 ± 0.4a	2.4 ± 0.1a	2.5 ± 0.5a	3.2 ± 0.2a
Vanillic acid	0.5 ± 0a	0.5 ± 0a	0.6 ± 0.1a	0.7 ± 0.1a	0.3 ± 0a	0.3 ± 0a	0.3 ± 0a	0.3 ± 0a
Oleacein	264.2 ± 18.8b	258.7 ± 20.4b	395.3 ± 24.4a	382.7 ± 19.1a	576.8 ± 26.5bc	562.2 ± 33.9c	671.8 ± 37.9a	645.7 ± 22.7ab
Oleocanthal	49.8 ± 8.4b	46.3 ± 0.9b	67.8 ± 14.6ab	73.1 ± 0.4a	137.9 ± 11.7a	130.9 ± 1.6a	162.2 ± 3.3b	161.6 ± 6.3b
(+)-1-acetoxypinoresinol	4.7 ± 0.2a	4.8 ± 0.1a	5.1 ± 0.5a	4.6 ± 0.3a	24.8 ± 1.6a	24.4 ± 0.9a	25.1 ± 0.8a	25.1 ± 1.1a
(+)-pinoresinol	7 ± 0.1a	6.6 ± 0.1a	7.3 ± 0.6a	7.1 ± 0.3a	10.3 ± 1.8a	10.4 ± 1.9a	10.6 ± 0.3a	10.2 ± 1.3a
Oleuropein aglycone	59 ± 4.2b	44.4 ± 2.7c	84.2 ± 3.8a	83.7 ± 4.9a	108.6 ± 3.9ab	106.8 ± 7.7b	129.8 ± 5.3a	121.6 ± 13.1ab
Ligstroside aglicone	8.2 ± 1.5b	6.4 ± 0.3b	11.7 ± 0.9a	12.2 ± 0.6a	20 ± 3.3a	22.6 ± 4a	19.1 ± 2a	20 ± 2.6a
Total phenols	396.1 ± 21.5a	370.7 ± 20.8a	575.1 ± 29b	569.5 ± 20.4b	883.1 ± 29.8b	861.6 ± 35.9b	1023.5 ± 38.8a	990.2 ± 30.1a
Oleuropein derivatives	324.2 ± 19.3b	304.3 ± 20.6b	480.9 ± 24.7a	469.7 ± 19.8a	687.4 ± 26.8bc	670.6 ± 34.7c	803.8 ± 38.3a	769.8 ± 26.2ab
Ligustroside derivatives	59.8 ± 8.6b	54.5 ± 1b	81.3 ± 14.7a	87.5 ± 0.8a	160.3 ± 12.1b	155.9 ± 4.4b	183.8 ± 4a	184.8 ± 6.8a
Lignans	11.7 ± 0.3a	11.4 ± 0.1a	12.4 ± 0.7a	11.7 ± 0.4a	35.1 ± 2.4a	34.8 ± 2.1a	35.7 ± 0.8a	35.3 ± 1.7a

Note: Results are the mean of two determinations in two independent experiments ± the standard deviation. Included in the sum of oleuropein derivatives are 3.4-DHPEA, 3.4-DHPEA-EDA, and 3.4-DHPEA-EA. Included in the sum of ligustroside derivatives are p-HPEA, p-HPEA-EDA, and p-HPEA-EA; in that of lignans are (+)-1-acetoxypinoresinol and (+)-pinoresinol. Different lowercase letters (a–c) indicate that the results are statistically different among theses for each different cultivar (*p* < 0.05).

**Table 3:** Volatile compounds (µg/kg) determined in the virgin olive oil samples from Peranzana (PE) and Coratina (COR) cultivars, obtained with traditional crushing (CR) and stoning (ST), with (O_2_) and without oxygen supply during extraction.

Volatile Compounds	Peranzana				Coratina			
	PE CR	PE CR-O_2_	PE ST	PE ST-O_2_	COR CR	COR CR-O_2_	COR ST	COR ST-O_2_
*Aldehydes*								
Pentanal	n.d.	n.d.	n.d.	n.d.	15 ± 2a	11 ± 3a	14 ± 7a	10 ± 1a
(*E*)-2-Pentenal	44 ± 4b	37 ± 4b	68 ± 16a	77 ± 5a	31 ± 2ab	23 ± 2b	47 ± 5a	40 ± 15ab
Hexanal	1052 ± 58a	969 ± 42ab	990 ± 13ab	912 ± 20b	1012 ± 85a	1006 ± 41a	818 ± 147a	902 ± 71a
(*E*)-2-Hexenal	15,358 ± 1111d	23,319 ± 915c	28,465 ± 896b	31,759 ± 1214a	38,908 ± 2650b	41,783 ± 1558ab	44,370 ± 1335a	45,895 ± 1006a
(*E*,*E*)-2,4-Hexadienal	247 ± 8b	268 ± 6b	308 ± 9a	300 ± 16a	174 ± 7a	160 ± 9a	176 ± 13a	178 ± 12a
*Ʃ of C_5_ and C_6_ aldehydes*	16,701 ± 1113d	24,592 ± 0c	29,832 ± 896b	33,048 ± 1214a	40,139 ± 2652b	42,984 ± 1559ab	45,425 ± 1343a	47,024 ± 1008a
*Alcohols*								
1-Pentanol	24 ± 2a	24 ± 1a	23 ± 1a	24 ± 1a	12 ± 2a	6 ± 3b	8 ± 1ab	7 ± 1b
1-Penten-3-ol	282 ± 22a	232 ± 15b	130 ± 4c	104 ± 2c	240 ± 19a	217 ± 17a	116 ± 11b	155 ± 13b
(*E*)-2-Penten-1-ol	20 ± 2a	18 ± 3ab	13 ± 4b	15 ± 1ab	26 ± 3a	22 ± 2ab	14 ± 1bc	12 ± 6c
(*Z*)-2-Penten-1-ol	240 ± 13a	201 ± 7b	199 ± 8b	169 ± 9c	242 ± 16a	239 ± 6a	173 ± 18ab	204 ± 32b
1-Hexanol	188 ± 14a	188 ± 12a	155 ± 2b	153 ± 5b	189 ± 13b	164 ± 11b	221 ± 8a	186 ± 14b
(*E*)-2-Hexen-1-ol	126 ± 8a	118 ± 8a	79 ± 21b	103 ± 8ab	477 ± 52a	475 ± 35a	493 ± 29a	460 ± 20a
(*Z*)-3-Hexen-1-ol	210 ± 16a	165 ± 10b	119 ± 14c	115 ± 12c	159 ± 17a	119 ± 8ab	82 ± 5bc	72 ± 29c
*Ʃ of C_5_ and C_6_ alcohols*	1091 ± 35a	947 ± 0b	717 ± 27c	683 ± 18c	1345 ± 62a	1242 ± 42a	1106 ± 37b	1095 ± 52b
*Esters*								
Hexyl acetate	180 ± 8c	229 ± 16b	307 ± 18a	305 ± 19a	57 ± 3a	57 ± 4a	59 ± 4a	57 ± 4a
(*Z*)-3-Hexenyl acetate	191 ± 14b	213 ± 6b	248 ± 9a	244 ± 11a	14 ± 0a	13 ± 2a	14 ± 1a	15 ± 3a
*Ʃ of esters at C_6_*	371 ± 16c	4421 ± 0b	556 ± 20a	549 ± 22a	71 ± 3a	70 ± 4a	74 ± 5a	72 ± 6a

Note: The data are the mean values of two independent extractions, ± the standard deviation. The values in each row with different letters (a–d) are significantly different for each different cultivar. n.d., not detected (*p* < 0.05).

## Data Availability

The original contributions presented in the study are included in the article, further inquiries can be directed to the corresponding author.
